# Canada’s Prosthetic Coverage: a Review of Provincial Prosthetic Policy

**DOI:** 10.33137/cpoj.v2i2.33489

**Published:** 2020-03-16

**Authors:** C.W. Howard, D.K. Saraswat, G McLeod, A Yeung, D Jeong, J Lam

**Affiliations:** 1 Cumming School of Medicine, University of Calgary, Calgary, Alberta, Canada.; 2 Faculty of Medicine & Dentistry, University of Alberta, Edmonton, Alberta, Canada.; 3 Max Rady College of Medicine, University of Manitoba, Winnipeg, Manitoba, Canada.; 4 Faculty of Medicine, University of Toronto, Toronto, Ontario, Canada.; 5 Faculty of Medicine, McGill University, Montreal, Québec, Canada.

**Keywords:** Prosthesis, Amputees, Amputation, Limb loss, Healthcare, Rehabilitation, Healthcare, Prosthetic coverage

## Abstract

The Canadian healthcare system serves as an example of equity and federal service to citizens across the world. However, it is not without its challenges. Prosthetic coverage across Canada is highly variable and largely unable to provide equal coverage for Canadian persons living with amputation. Many persons with limb loss are forced to rely upon personal resources, fundraising, or the charity of non-governmental organizations in order to meet this basic healthcare need. This disparity in the Canadian healthcare system is unusual and largely undescribed in the literature. We thus explore the nature of Canadian healthcare prosthetic coverage across Canada, investigating the variability in coverage, presence of prosthetic coverage policies, clarity of policy, eligibility criteria, and interval of prosthetic replacement. Our findings highlight potential areas for improvement within current Canadian healthcare policy.

## INTRODUCTION

Following loss of a limb, proper prosthetic treatment allows patients to perform activities of daily living and improves general health and wellness.^1^ Conversely, improper prosthetic care can lead to serious long-term complications and comorbidities including increased risk of falls, pain, and significant musculoskeletal and dermatological complications.^2^ Patients who lack access to prosthetic care entirely may become sedentary, exacerbating many comorbidities that are common in this population such as diabetes mellitus and cardiovascular disease.^3^

The average age-adjusted incidence of lower limb amputation in Canada was reported as 22.9 per 100,000 individuals, increasing over the years 2006-2011.^4^ According to estimates by the War Amputations of Canada, approximately 50,000 Canadians are living with limb loss.^5^ Amputation occurs due to a variety of causes, including complications of diabetes, vascular disease, infection, cancer, trauma, and congenital disorders.^6^ Regardless of the cause, patients living with amputation experience a loss in their daily functioning and face life-long physical and emotional challenges. Proper prosthetic care leads to improved functional outcomes, reduced comorbid disease and hospitalizations, and lower healthcare costs.^7^ Therefore, it is crucial to provide the optimal prosthesis for the patient.

Unfortunately, acquiring prosthetic limbs can be challenging for patients. A major barrier is the high costs of these devices, which includes not only the prosthetic components, but also the entire prosthetic treatment process, auxiliary parts for the limb, repairs of the limb, and eventual replacements.^5^ High costs may be driven by a low demand for prosthetic devices due to the relatively low incidence of amputations in the general population as well as the need to customize the prosthesis to each patient. Furthermore, the total cost of prosthetic care can vary greatly depending on the level of amputation and functional needs. Although objective Canadian data is unavailable, research from the United States’ Department of Veteran Affairs suggests that 5-year projected unilateral upper limb prosthetic costs range between $31,129 to $117,440, while 5-year projected lower-limb prosthetic costs range from $82,251 to $228,665 for veterans with limb loss.^8^

In Canada, healthcare is largely a provincial responsibility, with the exception of eligible Indigenous people, Canadian Forces personnel, veterans, inmates of federal prisons, and certain groups of refugees for whom the federal government is responsible for providing healthcare.^9^ To provide provincial healthcare, the provinces and territories created thirteen insurance plans, one for each province or territory. Notably, the Canada Health Act only requires provinces to cover hospital services, physician services, and surgical-dental services provided by hospitals, medical practitioners or dentists.^10^ While provinces may choose to cover further services, they are not required to do so, which has resulted in considerable variability between provincial service coverage.

In general, funding for prosthetic devices can come from federal or provincial programs, (including workers insurance for work related injury), private health insurance plans, philanthropic organizations such as the War Amputations of Canada or a combination of these sources. Often, a part of the cost is covered by a patient’s personal resources or through individual fundraising campaigns. Examining governmental coverage of prosthetic devices across Canada reveals considerable variation from province to province. We sought to assess these variations to determine the extent to which interprovincial access to prosthetic care in Canada is equitable, according to policy.

## METHODOLOGY

### Policy Review

Information was first gathered through communication with relevant stakeholders (listed in Table 1), such as: government officials involved with Pharmacare or the Ministries of Health; national and provincial representatives for persons with limb loss identified through conversation with the War Amputations of Canada; organizational leaders, such as the president of persons with limb loss associations; and prosthetists and physiatry specialists in amputation rehabilitation care, identified by communication with the aforementioned stakeholders. Initial conversations with stakeholders allowed a holistic approach to appraising prosthetic policies guided by values of stakeholders across the country. At least two sources were used for each province.

**Table 1:** Sources of prosthetic device coverage information by province and source type.

Province	Provincial Policy Document	Provincial Official	Clinician Contact (Physiatrist and/or prosthetist)	Person with Limb Loss Stakeholder
**BRITISH COLUMBIA**	PharmaCare,	PharmaCare	N/A	WarAmps Canada
	Prosthetic and Orthotic Program			
**ALBERTA**	Alberta Aids to Daily Living, General Policy & Procedures Manual	N/A	Glenrose Amputation Rehabilitation Program	Alberta Amputee and Sports Association
	Alberta Aids to Daily Living, Orthotic and Prosthetic Benefits		Community Prosthetic Facility	WarAmps Canada
**SASKATCHEWAN**	Saskatchewan Aids to Independent Living Program, General Policies	Extended Benefits, Drug Plan and Extended Benefits Branch, Ministry of Health	N/A	WarAmps Canada
**MANITOBA**	SMD Foundation,	Manitoba Health, Seniors and Active Living	N/A	WarAmps Canada
	The Assistive Technology Funding Guide			
**ONTARIO**	Limb Prostheses Policy and Administration manual	N/A	Ottawa Rehabilitation Centre	WarAmps Canada
**QUEBEC**	Schedule 1,	N/A	Amputee Program Institut de Réadaptation Gingras-Lindsay de Montréal	WarAmps Canada
	Tariff for Devices which Compensate for a Motor Deficiency and Insured Related Services			
**NEWFOUNDLAND & LABRADOR**	N/A	N/A	Regional Adult Rehabilitation, Palliative Care, and Geriatrics Program	WarAmps Canada
**NEW BRUNSWICK**	Social Development Prosthetic Program Policy	N/A	Institute of Biomedical Engineering/Atlantic Clinic for Upper Limb Prosthetics	WarAmps Canada
**NOVA SCOTIA**	Arm & Leg Prostheses Program, Prosthetists Guide	N/A	N/A	WarAmps Canada
**PRINCE EDWARD ISLAND**	Health PEI,	N/A	N/A	WarAmps Canada
	PEI Pharmacare Formulary			

Prosthetic device coverage information was then identified by review of provincial policy documents and provincial websites. We extracted the most up to date information relevant to coverage of prosthetic devices from the prosthetic provincial policy documents. Each document’s sections relevant to prosthetics; replacement; eligibility; advanced devices; and coverage were read. Specific phrases were searched using keywords: “3R106”, “12K42”, “knee”, “elbow”, “humeral”, “femoral”, “myo-“, “myoelectric”, “advanced”, “micro”, “microprocessor”, “eligible”, “eligibility”, “criteria”, “replacement”, and “year”. Information was then condensed and paraphrased to fit within Table 2.

**Table 2:** Traits of prosthetic coverage across the provinces. Policy documents were reviewed and details are reported to the extent which they are reported in policy documents. Coverage is defined as a percentage covered according to policy and procedure list of a basic upper prosthetic component (Ergoarm 12K42, 4552$), and a basic lower prosthetic component (Ottobock 3R106, 1923$).

Province	Coverage Basic Prosthetic	Replacement Interval	Coverage Advanced Prosthetic	Reference(s)
**BRITISH COLUMBIA**	Upper limb: 75%	3 years: general	Myoelectric coverage: no information	11,12
	Lower limb: 100%		Microprocessor knee: no coverage	
**ALBERTA**	Upper limb: 93%	2 years: basic functionality devices	Myoelectric coverage: requires pre-approval	13,14
	Lower limb: 100%	3 years: myoelectric arm	Microprocessor knees: grant up to $6000		
		5 years: microprocessor knee		
**SASKATCHEWAN**	Upper limb: 100%	3 years: general	Myoelectric coverage: case-by-case	15
	Lower limb: 100%		Microprocessor knees: up to $15 000	
**MANITOBA**	Upper limb: 100%	2 years: general	Myoelectric coverage: no information	16
	Lower limb: 100%		Microprocessor knees: no information	
**ONTARIO**	Upper limb: 75%	3 years: basic functionality devices	Myoelectric coverage: Up to 75% or maximum limit	17,18
	Lower limb: 75%	3 years: externally powered devices	Microprocessor knees: no information	
**QUEBEC**	Upper limb: 100%	Present, not further described	Myoelectric coverage: up to $8000, variable by product	19
	Lower limb: 100%		Microprocessor knees: no information	
**NEWFOUNDLAND & LABRADOR**	Upper limb: N/A	N/A	N/A	20,21
	Lower limb: N/A			
**NEW BRUNSWICK**	Upper limb: 100%	5 years: general	Myoelectric coverage: ineligible	22
	Lower limb: 100%		Microprocessor knees: maximum $20 000	
**NOVA SCOTIA**	Upper limb: 100%	4 years: adults	Myoelectric coverage: $5089 maximum	23
	Lower limb: 100%	2 years: children	Microprocessor knees: maximum $6511	
**PRINCE EDWARD ISLAND**	Upper limb: N/A	N/A	N/A	24
	Lower limb: N/A			

We compared four factors between provinces: patients’ eligibility for prosthetic coverage, the coverage available for basic prosthetic components, the coverage available for advanced prosthetic components, and replacement intervals. In our search, we defined a “basic prosthetic component” as a device not enhanced by myoelectric capability, micro-processing chips, or other features relying on onboard programming to modulate activity. Advanced prosthetic components were defined as any devices with myoelectric capability, micro-processing chips, or other electrical features.

For the purposes of this paper, only provincial health coverage was assessed, specifically including British Columbia, Alberta, Saskatchewan, Manitoba, Ontario, Quebec, New Brunswick, Nova Scotia, Prince Edward Island, and Newfoundland and Labrador. Health coverage in the territories, federal funding programs, and workers’ compensation insurance programs were not examined. Primary focus was placed on coverage within the province’s primary healthcare policy.

### Patient Eligibility Criteria for Prosthetic Coverage

Prosthetic policy documents for each province were reviewed for inclusion or exclusion criteria regarding patients qualifying for prosthetic device coverage. Further criteria for eligibility of prosthetic device funding were identified and compared.

### Basic Prosthetic Device Funding Comparison

We chose the Ottobock Ergoarm 12K42 (mechanical body-powered elbow joint), valued at $4552, and Knee 3R106 (pneumatic polycentric knee), valued at $1923, as representations of basic function prosthetic components due to their ubiquitous nature across healthcare funding schedules and being considered base-function by the prosthetic community. Wholesale costs as of July 10th, 2019 were obtained from a Canadian prosthetic retailer. However, these values do not account for additional lab fees charged for time, product, and skills, or for the prosthesis socket fitting (i.e. the value of the entire prosthetic device treatment). It is likely that the use of component prices as a surrogate for overall prosthetic coverage may result in an overestimation of funding coverage for prosthetic services. However, these methods yield insight into general funding policy trends across different policies. Funding catalogues and coverage plans for each province were investigated and used to compare the proportion of basic-level prosthetic component covered. Percentage of basic-level component covered was expressed as the wholesale cost of the prosthetic component divided by the maximum funding value or the maximum percentage covered as per prosthetic coverage policy.

### Advanced Prosthetic Device Funding Comparison

Prosthetic policy documents for each province were reviewed for any description of funding of advanced prosthetic devices. Due to variance in advanced prosthetic coverage policies, general policy for “advanced prosthetic devices” as described in policy documents were recorded. If further specification of advanced prosthetic device was made, the highest amount funded was documented. No standard advanced prosthetic component could be used to compare funding due to variance in prosthetic coverage policies across Canada.

### Replacement Interval

All prosthetic policy documents for each province were reviewed for frequency at which prosthetic devices could be replaced under their respective prosthetic programs. As all provinces allowed replacement upon medical need and review, only the typical interval of replacement was recorded. Any variation of typical replacement interval program was also reported.

## RESULTS

### Eligibility for coverage of prosthetic devices

We found eight provinces to have formal government policies regarding prosthetic device coverage (Table 1). In all the provinces offering governmental coverage of limb prosthetic devices, coverage eligibility requires that the device is medically necessary and prescribed as such by a certified medical practitioner (e.g. physician, nurse practitioner), and the device be fabricated and provided by a licenced prosthetist.^12,14–16,18,19,22,23^ However, in some provinces additional variable stipulations on whom is eligible to receive coverage beyond the prior mentioned baseline criteria. British Columbia, Saskatchewan, Manitoba, Ontario, and Prince Edward Island all require no additional governmental funding.^12,15–18,24,25^ Prince Edward Island also requires no membership with the Royal Canadian Mounted Police nor Canadian Armed Forces.^25^ Notably, in New Brunswick, patients must have demonstrated financial need and be registered with Social Development Health Services in order to be considered eligible for any prosthetic funding from the provincial government.^22^

Policies and procedures vary across the provinces, as do their clarity. For example, British Columbia requires pre-approval of a device to consider funding its cost, and requires pre-approval for device repairs over $400,^12^ although retroactive approval may be possible. Other provinces such as Alberta and Québec require patients access governmental programs outside of healthcare to receive funding.^14,19^ Within Alberta Aids to Daily Living, the governmental program which manages prosthetic devices, the bureaucratic process of funding a prosthetic device is outlined.^13^ However, this is not the case amongst the majority of provinces, and stakeholders anecdotally reported confusion in navigating governmental systems during our interviews.

According to our investigations, two provinces may not administer any government funding for prosthetic limbs. Prince Edward Island (PEI) does not employ a formal governmental coverage policy at the time of this writing;^24^ however, according to personal correspondence with a representative from Health PEI, basic model prosthetic devices may be covered in full by the PEI government, implying a case-based approval system. Additionally, Newfoundland and Labrador do not have an available prosthetic device policy,^20,21^ although correspondence with Eastern Health Newfoundland & Labrador has revealed that they provide case-by-case funding for those in financial need.

### Coverage of basic prosthetic devices varies widely by province

“Basic device” is a term which must be defined separately from basic function, as most provincial policies denote a mandate to provide devices which will achieve “basic functionality.” Despite this similar mandate, there is wide variation in the devices which are deemed necessary to achieve basic function. Some policies implicitly assert, via absence of funds for advanced components, that basic devices should always be sufficient to enable basic functioning. Other policies acknowledge, via funding availability, that advanced components may be needed to achieve basic functioning. This discrepancy may be partly due to different definitions of “basic function”, with some referencing activities of daily living, others instrumental activities of daily living, and others referencing ability to function and work more broadly. For example, Manitoba’s policy identifies a mandate to provide prosthetic devices to “assist in the basic activities of daily living”.^16^ Activities of daily living (ADLs) standardly refers to grooming, dressing, toileting, transferring/ambulating, and eating.^26^ British Columbia’s policy also contains a mandate to help patients “achieve or maintain basic functionality”,^11^ although this is defined on a case-by-case basis.^12^ Alberta uses a classification system similar to the U.S Medicare Functional Classification Level to determine whether a patient will benefit from a prosthesis and therefore whether they are eligible for funding for certain prosthetic components.^27^ Saskatchewan refers to activities of daily living in a broader sense including higher-functioning activities like physically-demanding gainful employment in manual labour.^15^

In addition to variation in definition of basic function, for provinces offering coverage of prosthetic limbs, the benefit limits by device vary widely, as demonstrated by our comparison of the degree of coverage for the Ottobock Ergoarm 12K42 elbow joint and Ottobock Knee 3R106 knee joint (Table 2). Direct province-to-province comparison was hampered by non-standard terminology and generally disparate policy approaches. Half of provinces achieved 100% coverage of both basic components: Saskatchewan, Quebec, Nova Scotia, New Brunswick, and Manitoba. Alberta achieved 93% coverage of the elbow and 100% of the knee. British Columbia achieved 75% elbow coverage and 100% knee coverage. Ontario achieved 75% coverage for both, up to a maximum benefit amount. Some provinces have alternative programs providing additional coverage for specific groups, such as those on social assistance due to disability or other causes (Table 2). Notably, Prince Edward Island and Newfoundland have no enshrined prosthetic policy, simply stating funding is determined on a case-by-case basis with no data regarding degree of coverage.

### Coverage of advanced prosthetic devices varies widely by province

Given the significant variability in advanced prosthetic coverage (Table 2), we highlight the differences. Ontario and Saskatchewan provide the most coverage for advanced prosthetic devices when deemed necessary for a given patient. Ontario offers $15,000 towards myoelectric upper limb devices, up to a maximum of $17,690 for select advanced components.^17,28^ Saskatchewan covers microprocessor knees up to $15,000, and considers myoelectric coverage amounts on a case-by-case basis.15 Alberta and Québec offer some additional funding for advanced components relative to basic components, but the benefit limits are in the $5,000 - $8,000 range, similar to those of basic devices.^14,19^ Alberta only contributes up to $6000 towards the cost of microprocessor knees, for example, and less for other advanced prostheses.^14^ Alberta policy also indicates that myoelectric upper extremity devices will be funded with prior approval after at least one year of body-powered prosthesis use, but without reference to other specific criteria.^13^ New Brunswick lies in the middle of these examples: the province offers up to $20,000 or $10,000 for above- and below-knee prostheses respectively and up to $10,000 for arm prostheses; however, myoelectric prostheses are explicitly excluded from this coverage.^22^

Unlike the aforementioned provinces, Manitoba and British Columbia provide no additional funding for advanced components. British Columbia and Manitoba may allow residents to select an advanced device in lieu of a basic device, with the benefit limit for the corresponding basic device applying.^12,22^ In both of these provinces the benefit limit for any device does not exceed $5,000,^12,16^ which may cover only a small proportion of the cost of an advanced device.

### Maintenance and repair of prosthetic device varies by province

Provinces differ in the replacement interval for prosthetic limbs, ranging from 2 years in Manitoba to 5 years in New Brunswick.^16,19,22^ Fortunately, claims for repairs and adjustments are considered throughout the device lifespan when necessary due to damage, a change in the patient’s medical condition, or growth.^12,14–16,18,19,22,23^

## DISCUSSION

### Coverage Eligibility and Availability

While most provinces allow all residents to access basic prosthetic care, significant shortcomings exist. Our review of existing policy documents details that only 30% of provinces have policy documents detailing eligibility criteria beyond basic requirements. There is no indication that the criteria are appropriately inclusive or restrictive, or if they were developed with the input of stakeholders. Notably, 20% of provinces require applicants to demonstrate financial need to access provincial funding for prostheses, while an additional 20% of provinces require demonstration of financial need to waive co-payments or co-insurances.

The criteria to demonstrate financial need can be restrictive and vary by province; for example, within New Brunswick a life insurance policy may count as an asset and thereby disqualify patients from financial assistance in purchasing a prosthetic device. Furthermore, 50% of provinces exclude a patient from receiving healthcare funding if they are eligible for funding from other governmental programs. These exclusions may lead to inequitable access to prosthetic devices and may prevent patients from achieving 100% coverage of a prosthetic device by combining coverage policies. Additionally, Newfoundland and Labrador and PEI have no publicly available documented coverage policy, and PEI coverage is organized within the Queen Elizabeth Hospital itself. Individuals in these provinces who are unable to afford prosthetic devices via personal means or private insurance may thus be denied the opportunity to receive them, resulting in inequitable access to prosthetic care.

### Variable and Insufficient Prosthetic Coverage

From our review of the provincial funding for prosthetic devices, we saw a wide range in the maximum funding available to cover various prosthetic components. Only 50% of the provinces surveyed had 100% coverage of both the upper and lower limb basic prosthetic components (Table 2). The degree of funding was variable across provinces, and it is notable that Alberta and Ontario require patients to cover at least 25% of their device, although in the case of Alberta, there is a maximum cost-share portion. In both of these provinces, patients with demonstrable financial need (such as receiving social assistance) can receive 100% coverage of basic prostheses. In provinces without coverage policies, it is impossible to know what proportion of value patients will pay as cases are considered on an individual basis. Comparing access to funding is difficult due to the variability in existing procedural policies for applying to receive funding for prosthetic devices, with some provinces having a defined procedure in place13 but other provinces such as Manitoba lacking procedural definition entirely.^13,16^ Given the high upfront cost of prosthetic devices and variable coverage policies, patients suffer either uncertainty or a significant cost burden, especially those who do not have alternative funding sources or personal savings.

For those provinces with a prosthetic coverage policy, there is a general aim to provide funding for “basic” functionality prosthetic devices; however, there is a lack of consistency between provinces on what constitutes basic functionality and which types of prosthetic devices may be necessary to achieve it. Not only are definitions variable, which results in variable prosthetic coverage, but the definitions can result in exclusive coverage; for example, while many provinces require no other governmental funding, Prince Edward Island will not cover members of the Royal Canadian Mounted Police nor Canadian Armed Forces (Table 2).^25^ Saskatchewan and Alberta currently lead as examples of clear definitions to guide funding devices that will return patients to their optimal functional level, not just provide minimal functionality, although correlating this classification method with the device funding actually received was beyond the scope of this review.

An additional source of provincial variation in coverage amounts may be the outdated nature of benefit schedules. For example, Ontario and BC benefit amounts appear to have been last updated in 2012.^12,18^ If benefit amounts are outdated, they may be insufficient to cover the full amount of current prosthetic devices on the market. If more current models of prosthetic devices are not listed in the funding schedule, they may not be covered. This is especially pertinent in the age of advancing prosthetic technology, in which new prosthetic devices may not be covered solely due to policy update neglect.

Provision of advanced prosthetic devices has been demonstrated to be cost-effective.^29-32^ Despite evidence of cost-effectiveness and higher levels of safety,^30^ provincial approaches to coverage of advanced prosthetic devices are variable. Coverage for devices with advanced functionality is generally more restrictive and fluctuant across Canada, with 40% of provinces having no coverage policy (Table 2). For provinces that do have a coverage policy, benefit amounts range from $5000 to $20,000. Given that a microprocessor knee may cost upwards of $40,000 to $45,000, even a maximum co-insurance funding amount leaves patients paying a significant portion personally, which may be impossible for middle-income earners who may not qualify for financial assistance but lack the resources to bear these costs on their own, further increasing the burden on patients.^17^ Furthermore, the success of funding requests and resultant provision of devices remains unknown, complicated by the case-based review system in some provinces with unstated criteria. Advanced prosthetic coverage remains limited, with the majority of provinces lacking coverage, provinces being highly particular in what prosthetic devices are covered, and coverage plans falling far short of total coverage.

### Variable Prosthetic Replacement Interval

Our work demonstrates that 50% of provinces have replacements offered every three years (Table 2). No difficulties with replacement interval were noted during our literature review, interviews with persons with limb loss, nor discussion with clinicians. However, difficulties may still arise. Prosthetics are at variable risk of degradation dependent upon prosthetic quality, activity level, and anatomic location. For example, a farmer utilizing a below-the-knee prosthesis daily may have significant wear and component failure within three years, prior to allowed replacement. This may lead high-activity and high functioning patients to suffer significant repetitive financial drain associated with repeat co-payment for necessary prosthetic maintenance.

### Coverage of Prosthetic Devices: Canada’s Equality

Prosthetic access and coverage should be based on need, irrespective of a patient’s identified province of residency. The described interprovincial inequality is unfortunately consistent with other growing healthcare inequalities across Canada.^33^ The widening gap in healthcare between provinces may be attributed to differential fiscal capacities of provinces, along with differing provincial government priorities.^34^ Differences in populations that comprise each province may also play a role, where there is a growing young population in Alberta while the aging population is on the rise in Maritime provinces, and variable prevalence of diabetes may result in proportionately variable prosthetic demands.^34^ The specific example of prosthetic coverage reveals the lack of a national standard as a contributing factor to the observed disparities between provinces. Altogether, this issue highlights the important need to establish a standard to allow for equal access to appropriate funding of prostheses across Canada.

A recent report released by the College of Family Physicians of Canada has outlined the responsibilities of the federal government as “providing adequate funding, establishing national standards, enforcing legislation, and ensuring all regions of Canada receive equal and appropriate resources”.^35^ Similarly, the World Health Organization (WHO) has recently advocated for the prioritization of universal health coverage for prosthetic and orthotic devices and services.^36^ Our work demonstrates that these goals are not being met in Canada. Importantly, the WHO has published an implementation manual for the standardization of prosthetic and orthotics services.^36^ This document provides a thorough summary of different domains which should be addressed and can serve as a valuable resource in the development of federal standards as advocated by the College of Family Physicians of Canada.^35^

Another area the WHO emphasizes is the accessibility of cost-effective prosthetic devices, even those which are deemed “sophisticated” or expensive. In Canada, there is notable resistance to the implementation of advanced prosthetic devices, as seen in the number of provinces which do not routinely fund devices such as myoelectric prostheses and microprocessor knees. Despite the higher costs of these devices, these types of prosthesis provide meaningful benefit to a patient’s quality of life and overall health. For example, the Canadian Agency for Drugs and Technologies in Health, a federal organization which reviews the evidence behind medical interventions, concluded in 2009 that there is cost benefit in the use of microprocessor knees,^37^ congruent with other work.^29–32^ In 2016 the National Health Service of England instituted a policy which provided coverage for microprocessor knees based on evidence of its cost-effectiveness.^38^ Currently, no provinces in Canada have a policy to fully fund these documented cost-effective devices.

### Limitations and Future Directions

Some inherent limitations are posed by the nature of this work. The lack of standardized policies across provincial coverage documents results in difficulty achieving comparability. While some provinces have extensively detailed lists of prosthetic devices which are funded and to what degree, other provinces may only list a handful, if any at all. Analysis of degree of coverage also relies upon knowing the cost of a given prosthetic component. However, the cost of a given prosthetic device is not readily available to the Canadian public, as most openly available information details government funding rather than specific market values. Similarly, the value of prosthetic devices we achieved in discussion with a Canadian prosthetics retailer represents wholesale cost, which means markup associated with skills and services cannot be accounted for, yet they are crucial components of adequate care and provision of devices. In addition, we analyzed only single components of the prosthetic device, not the entire prosthesis system that is required to treat a patient. This means we likely overestimate the degree to which provinces cover prosthetic devices, and therefore underestimate the degree to which funding of clinical services is necessary to ensure optimal outcomes.

It should be emphasized that the presented results are based on published policy, and do not take into account actual success rates of funding applications. In discussion with stakeholders, difficulty accessing full governmental funding was commonly stated as a barrier to selecting the right component for the individual patient. Future survey of Canadian prosthetic users, prosthetists, physiatrists, and government officials would be a useful endeavour to identify potential areas of policy development. For example, assessing the degree to which eligibility criteria or lack thereof have been problematic for prosthetic users across the provinces would validate the need for such policies. Given the lack of information regarding how prosthetic users fund their devices and to what degree they obtain less than ideal solutions due to funding limitations would yield crucial information in understanding the state of prosthetic coverage. Collection of such data would require a multi-institutional effort and would be a critical future direction.

## CONCLUSION

Funding for prosthetic devices at the provincial level should be updated and equalized across provinces to reflect the realities in the cost of prosthetic care and services. Failure to do so causes an unfair burden on the individual living with an amputation, often with dependence on geographic location. Emphasis should also be placed on providing the right prosthesis for the right patient, with the goal of restoring optimal function and reducing complications and comorbidities. Within an era of advancement of prosthetic technology, policy must adapt to ensure patients receive the best possible care.^39^ Without such changes, it is the persons with limb loss and their health that suffer the consequence of a system that has failed them. Our work has demonstrated the inability of the Canadian Healthcare System to provide both equitable and uniform prosthetic device coverage within all provinces, corroborating previous speculation.^39^ As such, the Canadian healthcare system has difficulty meeting the standards set both by itself and the WHO.^36,40^ Canada currently lacks uniform accessibility to prosthetic device coverage, uniform and equitable coverage of both basic function as well as advanced prosthetic devices, and uniform replacement intervals. Adequate coverage has not only been demonstrated to increase quality of life, but also to be cost-effective in the long-term.^30,31,41^ We have identified core deficiencies in prosthetic device care that should be addressed for the betterment of Canada and Canadian patients.

## DECLARATION OF CONFLICTING INTERESTS

The authors have no personal nor financial relationships to disclosure, nor any further conflicts of interest.

## AUTHOR CONTRIBUTION

- **Calvin W. Howard**,
  conceived the idea for the project, led data collection, and led manuscript writing.
- **Dave K. Saraswat**,
  conceived the idea for the project, led data collection, and led manuscript writing.
- **Graham McLeod**,
  assisted in manuscript preparation and data collection.
- **Albert Yeung**,
  assisted in manuscript preparation and data collection.
- **Danielle Jeong**,
  assisted in manuscript preparation and data collection.
- **Jack Lam**,
  assisted in manuscript preparation and data collection.

## SOURCES OF SUPPORT

This manuscript received no external supports.
